# Brucellosis Outbreak Traced to Commercially Sold Camel Milk through Whole-Genome Sequencing, Israel

**DOI:** 10.3201/eid2706.204902

**Published:** 2021-06

**Authors:** Svetlana Bardenstein, Rachel E. Gibbs, Yael Yagel, Yair Motro, Jacob Moran-Gilad

**Affiliations:** Kimron Veterinary Institute, Ministry of Agriculture and Rural Development, Beit Dagan, Israel (S. Bardenstein);; Ben Gurion University of the Negev School of Public Health, Beer Sheva, Israel (R.E. Gibbs, Y. Yagel, Y. Motro, J. Moran-Gilad)

**Keywords:** brucellosis, camel, milk, genomics, zoonoses, domestic, livestock, *Brucella melitensis*, bacteria, Israel

## Abstract

Brucellosis, a neglected zoonotic disease acquired from contaminated food products, remains a public health concern worldwide. We describe an outbreak in which commercially sold camel milk containing *Brucella melitensis* was distributed across Israel. Whole-genome sequencing linked patients infected with *B. melitensis* to wholesale camel milk and unregulated livestock trade.

Brucellosis, caused by bacteria of the *Brucella* genus, is a neglected zoonotic disease that affects marginalized populations worldwide (*1*). Human transmission occurs mainly through consumption of unpasteurized, contaminated dairy products from infected, domesticated animals. In Israel, brucellosis primarily affects Arab populations, especially the seminomadic Bedouin tribal communities in southern Israel (*2*).

Dromedary camels, which are capable of asymptomatic carriage of *Brucella*, are valuable domesticated animals in Bedouin culture, and unpasteurized camel milk has gained international popularity because of its alleged medicinal properties. Few reported brucellosis outbreak investigations involving camel milk have described families infected by milk from privately owned camels and have used serologic testing to confirm diagnoses (*3*,*4*). The standard tool for molecular typing of *Brucella* has been multilocus variable-number tandem-repeat analysis or multilocus sequence typing (MLST). However, whole-genome sequencing (WGS) is increasingly used for the study of genomic epidemiology of *B. melitensis* (*5*).

We report an outbreak of human brucellosis with unique epidemiologic characteristics, which originated from commercial, single-brand, unpasteurized camel milk; infection was diagnosed in 19 patients with a common exposure history over 4 months. We demonstrate the utility of WGS for brucellosis outbreak investigations.

## The Study

**Figure 1:**
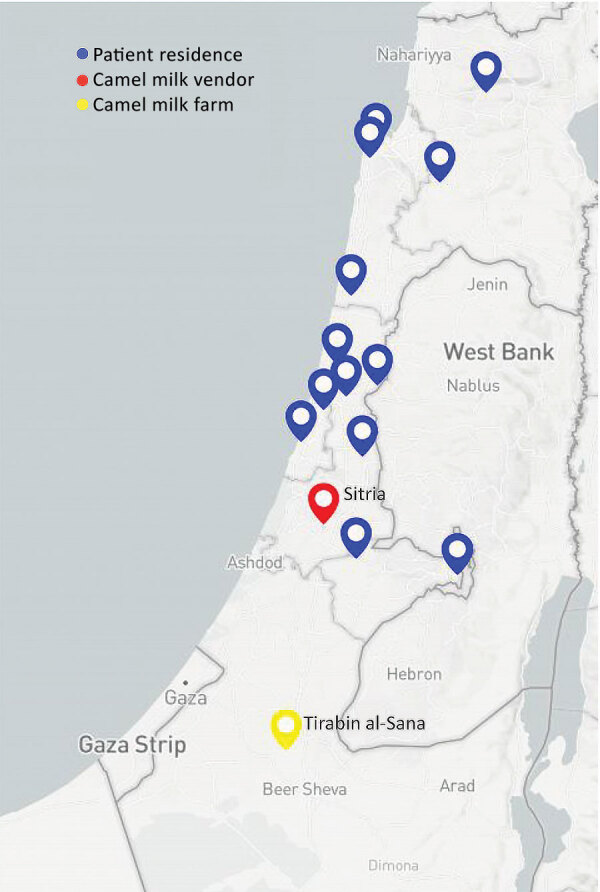
Geographic distribution of cases of *Brucella melitensis* traced to consumption of commercialized camel milk in Israel, 2016. Shown are the location of the camel farm in southern Israel from which raw milk was obtained), the vendor in central Israel that distributed the milk through direct online sales or other retail stores, and the places of residence of individual case-patients linked to the outbreak.

From July–November 2016, the Israeli Ministry of Health noted an increase in brucellosis cases in non-Arab patients in central and northern Israel, raising suspicion of a common source (Figure 1). An epidemiologic investigation noted patients were exposed to the same brand of camel milk. A total of 20 isolates were obtained from 19 patients across Israel (nos. 1–20; Table). Patients from shared households included 2 pairs of siblings and 1 married couple. We studied 2 isolates (nos. 1, 2) from an infant from whom *B. melitensis* was isolated from blood and cerebrospinal fluid. We also included 1 person (isolate no. 12) who consumed camel milk of an unknown brand.

**Table:** *Brucella melitensis* isolates included in the study of brucellosis outbreak traced to commercially sold camel milk through whole-genome sequencing, Israel*

Isolate no.	Sequenced	Origin	Age	Location	Relationship	Date of culture confirmation	Source
1	Yes	Human	Child	Jerusalem	Siblings	2016 Aug 30	Blood
2	Yes					2016 Aug 30	CSF
3	Yes	Human	Child	Jerusalem		2016 Oct 15	Blood
4	Yes	Human	Adult	Gan Haim	Married	2016 Jul 20	Blood
5	No	Human	Adult	Gan Haim		2016 Jul 20	Blood
6	Yes	Human	Child	Even Yehuda	Siblings	2016 Oct 7	Blood
7	No	Human	Child	Even Yehuda		2016 Jul 20	Blood
8	Yes	Human	Adult	Tel Aviv	None	2016 Aug 30	Blood
9	Yes	Human	Adult	Jerusalem	None	2016 Aug 14	Blood
10	Yes	Human	Adult	Herzeliya	None	2016 Oct 26	Blood
11	Yes	Human	Child	Elad	None	2016 Aug 3	Blood
12	Yes	Human	Adult	Tirat HaKarmel	None	2016 Nov 20	Blood
13	No	Human	Adult	Kochav Yair	None	2016 Aug 20	Blood
14	No	Human	Adult	Kochav Yair	None	2016 Aug 9	Blood
15	No	Human	Child	Ramat Yishai	None	2016 Sep 23	Blood
16	No	Human	Adult	Kochav Yair	None	2016 Jul 7	Blood
17	No	Human	Adult	Tzalfon	None	2016 Jul 15	Blood
18	No	Human	Adult	Carmiel	None	2016 Aug 8	Blood
19	No	Human	Adult	Haifa	None	2016 Aug 20	Blood
20	No	Human	Adult	Hadera	None	2016 Aug 10	Blood
21	Yes	Camel milk	NA	Food store	None	2017 Jan 15	Milk
22	Yes	Camel milk	NA	Food store	None	2017 Jan 15	Milk
23	Yes	Camel milk	NA	Food store	None	2017 Jan 15	Milk
24	Yes	Camel milk outlier	NA	Rahat	NA	2016 Jul 6	Milk
25	Yes	Human outlier case	NA	Taibe	NA	2016 Jul 11	Blood
26	Yes	Human outlier case	NA	Hebron	NA	2017	Blood
27	Yes	Human outlier case	NA	Haifa	NA	2017	Blood
28	Yes	Reference sequence (SRR4038984†)	NA	NA	NA	NA	NA

*CSF, cerebrospinal fluid; NA, not applicable.  †National Center for Biotechnology Information Sequence Read Archive database.

The suspected vendor obtained milk from a Bedouin camel farm in southern Israel (Figure 1). Field investigation of the farm revealed 32 female and 2 male camels. A total of 4 female camels had positive serologic test results for *Brucella*, but none were available for further testing. We sampled 6 bottles of camel milk obtained from a natural food store carrying the suspected brand and recovered a few colonies of *B. melitensis* from 3 of the bottles.

Clinical isolates were submitted to the National Brucellosis Reference Laboratory (Kimron Veterinary Institute, Beit Dagan, Israel) for confirmation, and milk samples were cultured at the same laboratory. In total, 10 *B. melitensis* human isolates from the outbreak and 3 camel milk isolates (nos. 21–23) from the implicated source were available for sequencing. An additional 4 epidemiologically unrelated isolates were sequenced and used as outliers, including 1 isolate from camel milk (no. 24), 2 isolates from patients with *B. melitensis* with no camel milk exposure (nos. 26, 27), and 1 isolate from an unrelated patient with *B. melitensis* acquired after consuming camel milk (no. 25). Finally, we used a *B. melitensis* reference genome sequence from the National Center for Biotechnology Information Sequence Read Archive database (no. 28).

DNA was extracted from *Brucella* isolates by heat killing (80 C°, 10 min) and by using the DNeasy Blood & Tissue kit (QIAGEN, https://www.qiagen.com). Genomic libraries were prepared with a Nextera Flex kit (Illumina, https://www.illumina.com) and subjected to paired-end sequencing by using the Illumina Miseq or Nextseq platforms. Sequences have been deposited in the European Nucleotide Archive (BioProject PRJEB43660).

We used WGS to analyze a total of 18 isolates: 10 human isolates, 3 camel milk isolates, 4 outlier isolates, and 1 reference sequence. Raw sequences underwent quality control using fastQC version 0.11.8 (*6*). We assembled sequence reads that passed QC by the pipeline shovill version 1.0.4 with Trimmomatic version 0.39, SPAdes version 3.13.1, and Pilon version 1.23 with the parameters “–trim –opts –sc” (*7*). We implemented in silico MLST with MLST version 2.10 per the pubMLST *Brucella* spp. Scheme (*8*,*9*). We conducted core genome single-nucleotide polymorphism (SNP) analysis of the WGS assemblies by using ekidna version 0.3.2 (*10*). The sequence from isolate 2 was selected for reference with complete assembly of 3,297,563 bases (Appendix Table 1). We masked resulting core genome SNPs for recombination events using Gubbins version 2.3.4 (*11*) and visualized the final SNP alignment as a minimum-spanning tree created by PHYLOViZ2 (*12*).

**Figure 2:**
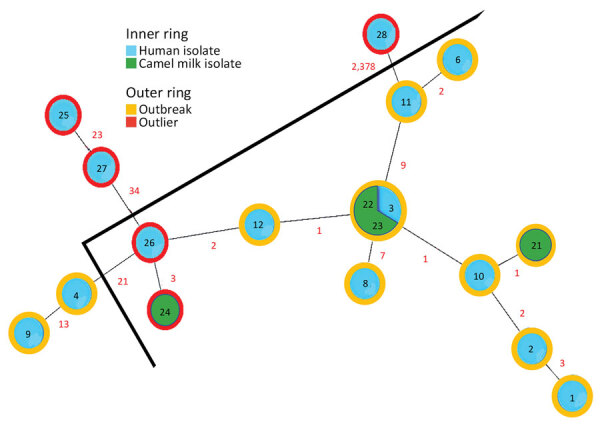
Minimum-spanning tree of core genome single-nucleotide polymorphisms analysis of *Brucella melitensis* outbreak traced to commercially sold camel milk, Israel, 2016. The phylogenetic tree includes human isolates, camel milk isolates, and both human and camel milk outlier sequences; numbers within circles correspond to isolate number given in the article text and numbers in red denote number of differing single-nucleotide polymorphisms between isolates. The nodes are colored according to the epidemiologic link (outbreak isolate or outlier), and outer rings are colored according to the sample source (clinical sample or camel milk).

We assigned all isolates to sequence type 8 by using in silico MLST. Core genome SNP analysis found 2,443 SNP sites with the sequence from isolate 2 for reference. The phylogenetic tree demonstrated 1 main cluster including 8 human outbreak isolates and all 3 isolates from camel milk (Figure 2). Two human outbreak isolates (nos. 4, 9) did not belong to the main cluster, suggesting >1 clone might have caused the outbreak. Isolate 12 clustered with outbreak isolates, confirming that the case-patient who consumed an unknown brand of camel milk was part of the outbreak. Outlier sequences (nos. 25, 27, 28) were genetically distinct from outbreak isolates, expectedly. However, outliers (nos. 26, 24) clustered with outbreak isolates, suggesting an unrecognized epidemiologic link between Bedouin camel farming and outbreak source.

## Conclusions

We describe an outbreak of brucellosis unique in its patient population, source of infection, chain of transmission, and use of WGS. Prior outbreak investigations of brucellosis have seldom used WGS to link the outbreak with samples from the implicated source. Here, WGS exposed genetic linkage between bottled camel milk and 8 isolates from 7 patients, providing conclusive evidence of this common source. Isolates obtained from the same person (nos. 1, 2) and that person’s sibling (no. 3) clustered tightly, providing internal validity to the discriminative power of WGS. WGS also associated isolate no. 12 (unknown camel milk brand) with the outbreak.

Two isolates (nos. 4, 9) from cases clustered apart from the outbreak strains. Because 4 female camels at the implicated farm had positive *Brucella* serologic test results, they might have harbored several different strains of *B. melitensis*, which could explain these results. We acquired all bottles of camel milk sampled during a single day, representing just 1 batch, and other batches were not tested. Therefore, the presence of additional clones implicated in this outbreak could not be ascertained.

Surprisingly, isolates 24 and 26 clustered with the outbreak strains, suggesting an unrecognized epidemiologic chain of transmission. This finding could reflect patterns of unregulated animal trade in Israel in which domesticated animals, including camels, are trafficked from Hebron (origin of isolate 26) throughout the Negev region to Bedouin communities (in this case Rahat [origin of isolate 24], the largest Bedouin city). Clustering of these outliers suggests that the outbreak might have been more widespread than documented.

In contrast with traditional patterns of brucellosis cases and case clusters, which occur mainly in small communities where residents consume locally produced unpasteurized dairy products, this outbreak resulted from web-based commercial sales of an unregulated food product. This sales channel enabled the spread of *B. melitensis* throughout Israel, similar to a recent United States report on commercially sold contaminated raw cow milk (*13*). Therefore, modern consumer trends in small agriculture and food production may result in new food safety risks.

The advantages of WGS in this outbreak investigation included the capacity to analyze both related and outlier isolates. This capability enabled us to resolve complex linkages between cases, suggesting unequivocal, temporal relationships that depict outbreak directionality and can aid future preventative measures.

In conclusion, we describe a unique *B. melitensis* outbreak linking human cases with commercially sold, unregulated camel milk. These results demonstrate that WGS can be a powerful tool for investigating transmission of brucellosis, a neglected zoonotic disease, in a world of modernized commercialism.

Appendix Additional info about brucellosis outbreak traced to commercially sold camel milk through whole-genome sequencing, Israel.

## References

[1] Pappas G, Papadimitriou P, Akritidis N, Christou L, Tsianos EV. The new global map of human brucellosis. Lancet Infect Dis. 2006;6:91–9. 10.1016/S1473-3099(06)70382-6 16439329

[2] Anis E, Leventhal A, Grotto I, Gandacu D, Warshavsky B, Shimshony A, et al. Recent trends in human brucellosis in Israel. Isr Med Assoc J. 2011;13:359–62. 21809734

[3] Shimol SB, Dukhan L, Belmaker I, Bardenstein S, Sibirsky D, Barrett C, et al. Human brucellosis outbreak acquired through camel milk ingestion in southern Israel. Isr Med Assoc J. 2012;14:475–8. 22977965

[4] Garcell HG, Garcia EG, Pueyo PV, Martín IR, Arias AV, Alfonso Serrano RN. Outbreaks of brucellosis related to the consumption of unpasteurized camel milk. J Infect Public Health. 2016;9:523–7. 10.1016/j.jiph.2015.12.006 26796768

[5] Tan K-K, Tan Y-C, Chang L-Y, Lee KW, Nore SS, Yee W-Y, et al. Full genome SNP-based phylogenetic analysis reveals the origin and global spread of Brucella melitensis. BMC Genomics. 2015;16:93. 10.1186/s12864-015-1294-x 25888205 PMC4409723

[6] Andrews S. FastQC: a quality control tool for high throughput sequence data. 2018 [cited 2021 Apr 4]. https://www.bioinformatics.babraham.ac.uk/projects/fastqc/

[7] Seeman T, Kwong J, Gladman S, Goncalves da Silva A. Shovil. Faster SPAdes assembly of Illumina reads. [cited 2021 Apr 4]. https://github.com/tseemann/shovill

[8] Jolley KA, Bray JE, Maiden MCJ. Open-access bacterial population genomics: BIGSdb software, the PubMLST.org website and their applications. Wellcome Open Res. 2018;3:124. 10.12688/wellcomeopenres.14826.1 30345391 PMC6192448

[9] Seeman T. mlst Github [cited 2021 Apr 4]. https://github.com/tseemann/mlst

[10] Seeman T. Ekidna [cited 2021 Apr 4]. https://github.com/tseemann/ekidna

[11] Croucher NJ, Page AJ, Connor TR, Delaney AJ, Keane JA, Bentley SD, et al. Rapid phylogenetic analysis of large samples of recombinant bacterial whole genome sequences using Gubbins. Nucleic Acids Res. 2015;43:e15–15. 10.1093/nar/gku1196 25414349 PMC4330336

[12] Nascimento M, Sousa A, Ramirez M, Francisco AP, Carriço JA, Vaz C. PHYLOViZ 2.0: providing scalable data integration and visualization for multiple phylogenetic inference methods. Bioinformatics. 2017;33:128–9. 10.1093/bioinformatics/btw582 27605102

[13] Gruber JF, Newman A, Egan C, Campbell C, Garafalo K, Wolfgang DR, et al. Notes from the field: Brucella abortus RB51 infections associated with consumption of raw milk from Pennsylvania—2017 and 2018. MMWR Morb Mortal Wkly Rep. 2020;69:482–3. 10.15585/mmwr.mm6915a4 32298248 PMC7755062

